# Vascular normalisation as the stepping stone into tumour microenvironment transformation

**DOI:** 10.1038/s41416-021-01330-z

**Published:** 2021-04-07

**Authors:** Anette L. Magnussen, Ian G. Mills

**Affiliations:** 1 Nuffield Department of Surgical Sciences, University of Oxford, John Radcliffe Hospital, Oxford, UK; 2 Patrick G Johnston Centre for Cancer Research, Queen’s University of Belfast, Belfast, UK; 3 Centre for Cancer Biomarkers, University of Bergen, Bergen, Norway; 4 Department of Clinical Science, University of Bergen, Bergen, Norway

**Keywords:** Tumour angiogenesis, Cancer microenvironment

## Abstract

A functional vascular system is indispensable for drug delivery and fundamental for responsiveness of the tumour microenvironment to such medication. At the same time, the progression of a tumour is defined by the interactions of the cancer cells with their surrounding environment, including neovessels, and the vascular network continues to be the major route for the dissemination of tumour cells in cancer, facilitating metastasis. So how can this apparent conflict be reconciled? Vessel normalisation—in which redundant structures are pruned and the abnormal vasculature is stabilised and remodelled—is generally considered to be beneficial in the course of anti-cancer treatments. A causality between normalised vasculature and improved response to medication and treatment is observed. For this reason, it is important to discern the consequence of vessel normalisation on the tumour microenvironment and to modulate the vasculature advantageously. This article will highlight the challenges of controlled neovascular remodelling and outline how vascular normalisation can shape disease management.

## Background

The vascular system spans about 100,000 miles within the human body, distributing oxygen and essential nutrients to organs and cells and taking away the by-products of metabolism.^1^ Ever since William Harvey first described this system in 1628, much has been written about this amazing network of arteries, veins and capillaries.^2^ Furthermore, one of the first scientific reports that documented a systematic approach observing the architectural changes in the vascular system after external stimuli date back as early as the 1850s.^3–6^

Vessels are generated by two distinct mechanisms. In early development, vasculogenesis involves the de novo formation of blood vessels by endothelial precursor cells (EPCs), which follow cues from growth factors and cytokines to create a primitive vascular tree; this vascular tree then gets remodelled and expanded by another mechanism, angiogenesis.^7–9^ However, EPCs from the bone marrow are known to participate in normal and pathological vessel formation in an adult.^10,11^

In healthy adults, endothelial cells tend to be quiescent and their turnover is very low compared with cells from other organs, for example, the gut. With the exception of the female reproductive cycle, angiogenesis is almost always pathological and the result of a trauma, surgery or an illness, such as cancer. Cancer cells are able to grow into solid tumours only by procuring a supply of nutrients and oxygen to meet their increasing demands for energy. They achieve this by creating a blood supply, either by exploiting the existing vasculature through co-option or vascular mimicry and/or by coaxing the existing vessels to expand through angiogenesis. As mentioned before, bone marrow-derived EPCs are also able to contribute to new vessel formation in an adult, although it is difficult to determine the exact extent of their contribution towards the tumour vasculature;^12–14^ some indication can be gained by indirectly assessing the number of cells that circulate in the blood.^15^

**Fig. 1:**
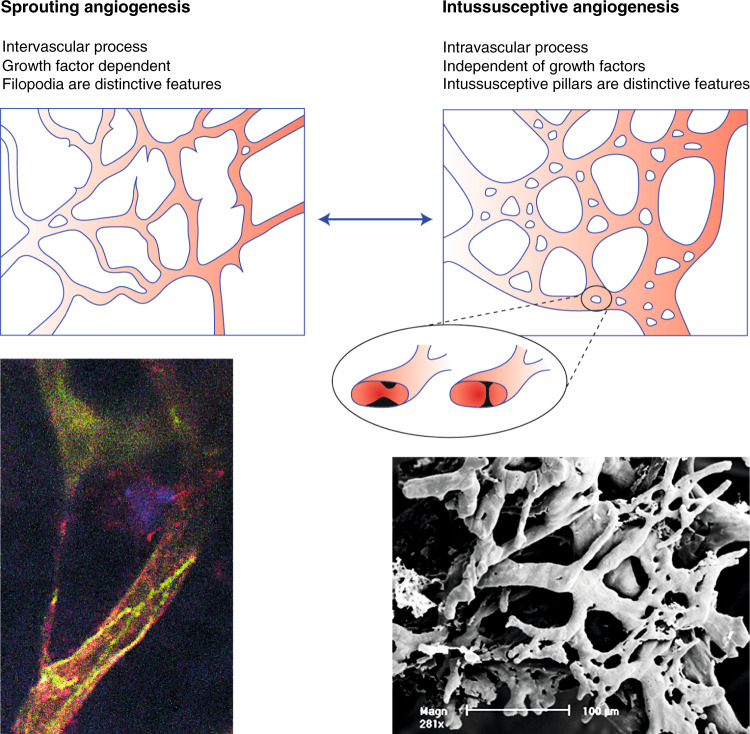
Schematic illustration of the vascular network undergoing sprouting or intussusceptive angiogenesis. The two mechanism of angiogenesis are not always exclusive, and a tumour can present characteristics of both, especially after anti-angiogenic therapy. On the left the cartoon illustrates how endothelial filaments called sprouts extending into the extravascular space. The accompanying confocal microscope image shows a filamentous bridge between two capillaries across the extracellular space. The endothelial cells and filaments are positive for PECAM-1 (green) and α5β1 integrin (red) in the Rip-Tag2 mouse model of pancreatic cancer. The cartoon on the right magnifies the cylindrical microstructures spanning the lumen of a capillary that are the distinctive feature of intussusception. For reasons not well understood endothelial cells on opposite sides on the lumen start to bulge until they meet to create the intussusceptive pillar. In the scanning electron micrograph on the right, those pillars appear as tiny holes on the outer surface on the vessel. The vascular casts of a colon tumour xenograft model reveal those very characteristic tiny holes of intussusceptive angiogenesis in the larger capillaries. The small diameter of ≈3–5 µm exclude them as mesh-structure in the vasculature. At this point, intussusceptive angiogenesis seems favoured over endothelial cell sprouting in those capillaries.

The majority of solid tumours are sustained by sprouting angiogenesis, in which endothelial cells can become tip cells or stalk cells (Fig. 1). Not all tumour growth necessarily depends on this mechanism. Intussusceptive angiogenesis is a less frequent, non-sprouting mechanism to increase vessel density that splits the existing vessel longitudinally, in principle dividing the lumen. Intussusceptive pillar formation is the defining feature (Fig. 1). There is little endothelial cell proliferation in the early stages of intussusceptive angiogenesis and pillar formation appears independent of angiogenic factors. There is no distinct growth factors gradient that guides the pillar forming endothelial cells in the way growth factors guide filopodial protrusions of a tip cell.^16^ This independence of growth factors can be a bypass around typical modes of anti-angiogenic targeting before sprouting angiogenesis prevails again.^17,18^

The fact that the circulation is required for tumour growth was known before the expression ‘tumour angiogenesis’ was coined.^19,20^ Evidence that existing host vessels are indeed seeding new vasculature following an activation signal and begin to sprout emerged with Judah Folkman’s ground breaking discovery in 1971 of a ‘tumour angiogenesis factor’.^21^ The theory had been that if a cancer could be stopped from growing its own blood supply it would wither and die. We now know that a ‘tumour angiogenesis factor’ does not exist in this simplistic form. Rather, a plethora of growth factors, together with other cytokines and biomolecules of various shapes and sizes, are involved in the process of angiogenesis (even different mechanisms of angiogenesis itself),^22–25^ such that targeting only one of the factors or receptors often spontaneously leads to the upregulation or activation of another factor to compensate for the loss.^26^ We also know that an intact, functional vascular system constitutes a key systemic route for the delivery of drugs and in particular is required for the responsiveness of the tissue microenvironment to these, and other, therapeutic agents, especially in the case of the tumour microenvironment (TME). Biologically and mechanistically treatment resistance is best understood in the context of tumour hypoxia. In the absence of a functional vascular network oxygenation of the TME is insufficient hence rendering chemotherapy, radiation and photodynamic therapy less efficient and the success of the latter depends on the generation of reactive oxygen species (ROS).^27,28^

Thus, targeting the blood supply of a tumour presents a challenge (Box 1). What has emerged from the study of the vasculature in response to anti-angiogenic drugs is that these agents can induce the phenomenon of vascular normalisation. This phenomenon can be exploited to confer improved efficacy in the course of anti-cancer treatments. In this article, we review the factors that regulate the vasculature and approaches to distinguishing between normalised and impaired vascular function. We conclude by proposing that temporal and spatial monitoring of vascular function will be of critical importance to maximising treatment impact and suggest ways in which this could be achieved in the future .

Box 1 **Endothelial cell heterogeneity**

The vascular system can be classified according to size, function, cell morphology, gene and protein expression, depending on the organs that are supplied—in other words, on the nature of the vascular bed. Arteries, veins, arterioles and venules are larger vessels that narrow into a network of capillaries. The larger vessels consist of a continuous endothelium, held together by tight junctions, with three strong outer support layers. Capillaries are fine and narrow vessels that can be continuous, fenestrated or discontinuous, and are stabilised by the basal lamina and pericytes. The phenotypes of endothelial cells vary with their function and vascular bed. Extreme examples would be the highly fenestrated or discontinuous endothelium of capillaries in filtrating organs, like the kidneys and liver, and the tight endothelium of capillaries in the brain that constitutes the blood–brain barrier.^146^ Endothelial cells are capable of sensing and responding to their environment, which accounts for their phenotypic heterogeneity. Separated from their vascular bed and cultured in situ, they undergo a phenotypic drift. DNA microarrays reveal that about 50% of their site-specific genes are lost after passage. Concurrently, some specific properties are retained under cell culture conditions, which means that their gene expression is mediated by both their immediate environment and by epigenetics.^147^

**Endothelial cell heterogeneity in tumour vessels**

The phenotype of tumour endothelial cells (TEC) differs greatly from the normal endothelial cells in the host organ. The epigenetic footprint of endothelial cells in the tumour vasculature, however, is conserved, meaning the specific properties linked to their original vascular bed are kept.

For a long time, it was accepted that TEC cells within tumours were genotypically equal to their normal counterparts and that any changes in phenotype were a mere reaction to the cancerous environment. Today it is appreciated that TECs can display cytogenetic abnormalities. TECs derived from malignant melanoma and liposarcoma are not normal diploid cells but contain an abnormal number of chromosomes in the nucleus (aneuploidy). The chromosomal instabilities are not clonal but heterogeneic within a TEC population. However, the mechanisms that cause TEC heterogeneity are still pinned on the tumour environment.^62,148^

## Tumours contain abnormal blood vessels

**Fig. 2:**
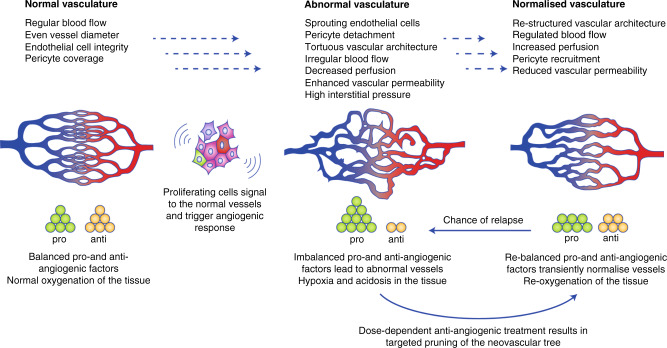
Schematic illustration of the vascular network in its normal, abnormal and normalised state. Angiogenic and anti-angiogenic factors are finely tuned in healthy tissues to create an organised vessel structure and to maintain vascular function. In tumours, the angiogenic switch has taken place and the balance has tipped in favour of angiogenic factors. As a result, the structure of neovessels is abnormal on all levels, with highly impaired vascular function. In the normalised state, angiogenic and anti-angiogenic factors are nearly balanced and vascular function is transiently re-established.

Whichever of the mechanisms that a solid tumour uses for increasing the blood supply (and, in many cases, a combination to various degrees of all seems the reality), the newly formed vessels (neovessels) are abnormal in the majority of cases.^29,30^ A relentless exposure to angiogenic factors released from the hypoxic tumour environment renders the endothelial cells in a state of constant activation, resulting in an uneven and dilated lumen and a tortuous vessel architecture (Fig. 2). Despite a seeming abundance of blood vessels, many of which retain blood flow, large areas within the tissues remain hypoxic. Chronic hypoxia stems from the shortened radial oxygen diffusion distance in tumour blood vessels. Moreover, irregular blood flow and fluctuation in perfusion adds acute or ‘cycling hypoxia’ to the overall chronic hypoxic state of the tumour.^31^ Mural cells called pericytes on capillaries are no longer adherent, and thereby lose their regulatory influence on the vessel stability.^32^ The basement membrane becomes thickened in some places and is absent in others.^33^ Weak endothelial cell–cell junctions in the tumour vasculature enable rogue cancer cells to enter the circulation—the first step of potential metastasis^34^—as well as creating gaps and leaky vessels. Although vessel leakiness might improve drug delivery by facilitating the passage of molecules through the endothelial lining,^35^ this benefit is offset by restricted blood flow and high interstitial pressure in the tumour. The lack of a pressure gradient shifts the distribution mechanism (of particles in general) from being directional (movement from a high-pressure region to a low-pressure region) to being non-directional (‘diffusion’). Diffusion through the dense extracellular matrix (ECM) is extremely slow so that some macromolecules can be re-absorbed into the circulation. This has been exemplified experimentally for globular IgG, albumin and dextrans using fluorescence correlation spectroscopy.^36,37^

## Angiogenesis inhibitors and vascular normalisation

The rationale behind anti-angiogenic treatment is that blocking blood vessel formation in tumours or its regression will deprive cancer cells of nutrients and oxygen and finally starve tumours to death or induce tumour dormancy. However, anti-angiogenic treatment fails to bring about permanent de-vascularisation; rather, normalised tumour vessels emerge from the neovascular tree. Drug-induced vessel normalisation was observed and documented in 1972 by Le Serve and Hellman, when treatment with ICRF-159 (razoxane) was seen to cause the chaotic tumour neovasculature of Lewis Lung Cancer (LLC) in mice to appear normal.^38^ The drug was discovered through random screening and selected for its cytotoxic properties. At the time, it was largely unknown how ICRF-159 changed the architecture of the tumour vessels. Today we know that ICRF-159 is an inhibitor of the platelet-derived growth factor-β (PDGF-β) receptor, and its anti-angiogenic properties and potential as a vessel normalisation drug have been established.^39^ The response of the pathological neovasculature to anti-angiogenic treatment had not been anticipated and the effect that normalisation has on malignant and non-malignant cells opened new avenues for cancer combination therapy.^40–42^

Vessel normalisation almost always follows the same recognisable pattern in terms of vascular structure plasticity and changes to the TME.^43^ The immature parts of the neovasculature seem to be more susceptible to anti-angiogenic treatment than more mature vessels are, indicating that mature vessels are evidently less dependent on angiogenic factors. Tumour vessels that do not regress after anti-angiogenic treatment appear normalised, as illustrated in Fig. 2. The reduced cellular demand for blood supply re-establishes the equilibrium between angiogenic and anti-angiogenic factors. During the following vessel stabilisation effect, the endothelial cells in the immature vessels secrete basement membrane proteins and send signals that recruit mural cells. Cell junction integrity is resumed and permeability is again tightly regulated.^44^ However, the most important biological aspect of normalised vessels is that a reliable circulation replaces the previously flawed system, such that restored perfusion reverses almost all the abnormalities.

### Assessing normalised vessels

In animal models, vessel normalisation is mostly observed by microscope image analysis, either at fixed time points or in real time (via a dorsal window chamber).^45,46^ Contemporary image analysis software allows for the quantitative analysis of vessel branching, number of sprouting endothelial cells, vessel length and diameter. Spatial image analysis of distance, for example, between pericytes and the endothelium is an indicator of vessel maturity. Blood flow and perfusion can be monitored by systemic optical tracers or non-invasively by functional magnetic resonance imaging (fMRI) and photoacoustic imaging.^47,48^ Visual and functional assessment of vascular normalisation is usually supported by complementary analysis at the molecular level, such as the analysis of the expression of angiogenic factors.

We can define normalised vessels according to several prominent characteristics. A significant drop in angiogenic factors results in fewer sprouting endothelial cells. Mural cells, such as pericytes, adhere again to the vessel walls and pericyte coverage increases. Through remodelling and reduced branching, leaner and less tortuous vessels with an even luminal diameter emerge, reducing the blood back flow that is typical for abnormal vessels and restoring a uniform blood pressure. Pruning of truncated vessels and endothelial structures that lack a lumen improves the perfusion. In the surrounding tissue, oxygen levels rise and hypoxia decreases, which, in turn, reduces hypoxia-induced activation of angiogenesis. In short, blood vessel maturation takes place.^49^ In reality, a combination of these restored normal blood vessel characteristics usually appears, with degrees of intra and inter tumour variation, which might depend on the tumour type and treatment.

## Current strategies and potential targets for tumour vessel normalisation

**Fig. 3:**
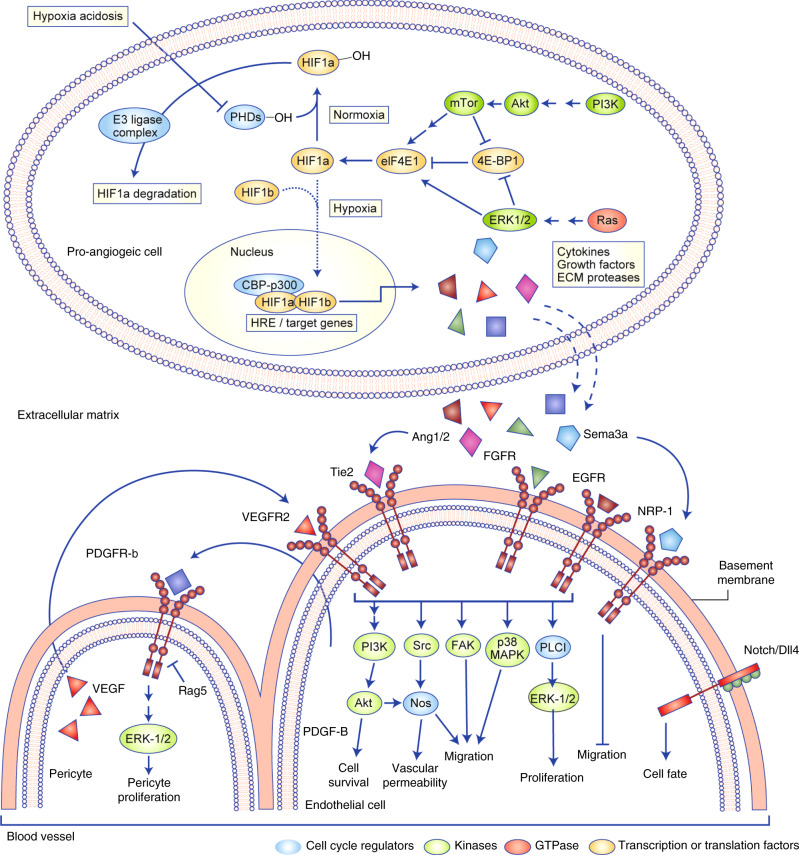
Illustration of the major signalling pathways that either control angiogenesis and therefore represent potential targets for anti-angiogenic intervention. In normal tissues, prolyl hydroxylases (PHDs) hydroxylate hypoxia-inducible factor (HIF)-1α; the level of HIF-1α is low or it gets degraded. Hypoxia, however, prevents the hydroxylation and HIF-1α levels rise. The HIF-1α/β complex binds to the hypoxia-response element (HRE) of the gene promoter for transactivation and expression of angiogenic factors, prominent among which are VEGF/VEGFR-2 and other activated tyrosine kinase receptors that determine cell survival, proliferation and cell motility. Tie2 and Ang-1 are vital for maturation and maintenance of the neovessels. Ang-2 in combination with VEGF promotes neovascularisation, but in the absence of VEGF triggers vessel regression. Neuropilin-1 is the specific receptor for class 3 Semaphorin (Sema) proteins. The anti-angiogenic Sema3/neuropilin complex controls endothelial cell motility by negatively regulating cytoskeletal events via small GTPase signalling. VEGF-dependent Notch-1/DLL4 receptor activation specifies endothelial cell fate. In cells with high Notch-1 activation the angiogenic potential is lowered, those cells remain quiescent, in cells with low Notch-1 activation the angiogenic potential is raised and they become sprouting cells. The pathways that regulate angiogenesis are interlinked.

The signalling pathways that lead to sustained angiogenesis are illustrated in Fig. 3, and include several different types of molecule, such as kinases and GTPases. Targeting any of these molecules is likely to bring about changes in the vasculature that might influence tumour vessel normalisation. Similarly, promoting factors that initiate vascular maturation and sustain maintenance can induce vascular normalisation.

### Inhibiting proangiogenic signalling

**Fig. 4:**
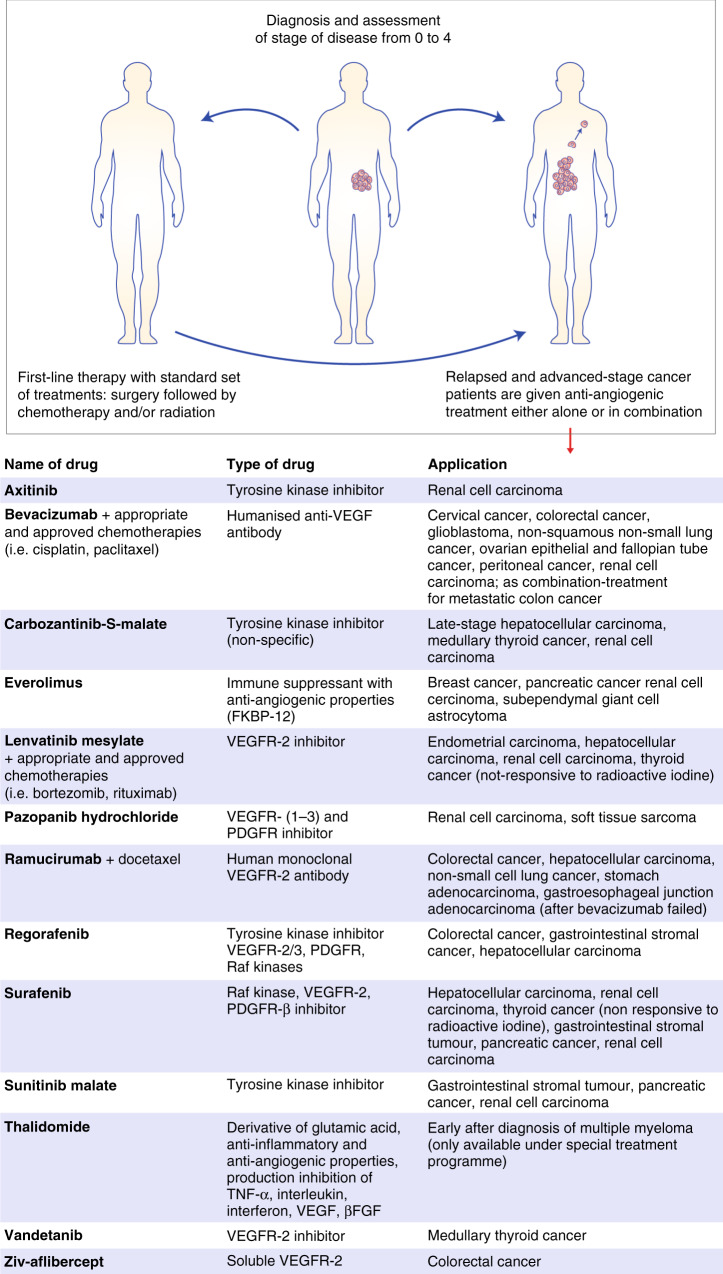
Anti-angiogenic treatment is approved by the FDA for the treatment of advanced cancers after conventional chemotherapy has failed or if cancer has metastasised (last reviewed 2019 source NIH, cancer.gov). Only three anti-angiogenic dugs are applied in combination with traditional chemotherapy (bevacizumab, lenvatinib mesylate, ramucirumab). Thalimodide and lenvatinib mesylate are the only two treatments that are used in the early stages following diagnosis or as a first-line treatment and if surgery is not an option. The vast majority of anti-angiogenic drugs are tyrosine kinase inhibitors with some of them acting specifically on the VEGFR-2 receptor.

As represented in the table in Fig. 4, targeting VEGF-A with blocking antibodies or using soluble VEGF receptors, or directly inhibiting the VEGF-receptor 2 (or, less specifically, tyrosine kinase receptors in general) was, and still is, the first choice to downregulate neovascularisation.^39^ Early research to prove the hypothesis of vessel normalisation used DC101, a monoclonal antibody against VEGFR-2. A single intravenous dose of DC101 administered to tumour-bearing mice could reduce the interstitial fluid pressure by 50% without evidence of lymphatic vessel restoration.^46^ Extensive vascular pruning of vessels in the tumour was observed through a dorsal window chamber. Vascular density dropped and vessel diameter shrunk. In addition, metalloproteinases that degraded layers of the basement membrane to a normal thickness were activated, and levels of angiopoietin-1 (Ang-1), which supports the recruitment of new pericytes, increased temporarily. Another important finding from these investigations was that the vascular normalisation effect was transient and occurred 3–5 days post treatment, and that, during this time, the response to radiation therapy was greatly improved.^50,51^ These findings gave rise to the idea that, rather than inhibiting angiogenesis altogether, controlling angiogenesis would be a preferable approach.

VEGF is highly expressed in hypoxic areas. The ectopic expression of a recombinant soluble form of VEGFR-2 in melanoma cells under hypoxic conditions did indeed bind murine and human VEGF sufficiently to slow down endothelial cell growth in vitro and reduce the tumour mass in vivo,^52^ and increased oxygen levels and reduced hypoxia suggested normalisation of the vessels. Despite controversy about the biological properties and function of VEGFxxx isoforms they were reported to promote angiogenesis while VEGFxxxb isoforms (generated by alternative splicing) have been shown to inhibit angiogenesis.^53^ Re-establishing the natural equilibrium of angiogenic and anti-angiogenic VEGF-A splice variants might offer an alternative means of tuning the angiogenic switch instead of blocking it. Although this method of regulation was achieved by locally adding the anti-angiogenic variant, modulation on a transcriptional level by inhibiting SRPK-1, the key factor in the spliceosome that regulates VEGF-165/VEGF-165_b_ expression might be a more promising approach in the future.^54–56^ The regulatory effect of the VEGF-165_b_ variant on the neovasculature is considered particularly advantageous in diabetic retinopathy, where a normalised vascular network is preferable over a significant de-vascularisation.^57^

Angiogenesis is regulated by a complex network of direct and indirect factors. In many ways, these factors are not entirely independent of VEGF/VEGFR-2 signalling. Some of them bear, for example, a resemblance to VEGFR because they are also tyrosine kinase receptors and signal via the same pathways, and/or trigger VEGF upregulation or otherwise influence the VEGF/VEGFR-2 pathway further downstream. Although it mediates downstream signalling by inducing serine/threonine kinase activity, rather than by activating a tyrosine kinase receptor, transforming growth factor β (TGF-β) plays a role in tumour progression.^58,59^ Blocking TGF-β and its receptors decreased the tumour size and prevented metastasis in breast cancer and glioblastoma models and significantly improved the intratumoural penetration of low-molecular weight chemotherapy drugs and nanoparticles in breast cancer models. The effect was attributed to tumour vessel normalisation, as a higher number of perfused vessels was counted and pericyte recruitment to these vessels was observed.

Epidermal growth factor receptor (EGFR), a tyrosine kinase receptor that, among several other functions, activates H-Ras to stimulate angiogenesis, and phosphoinositide 3-kinase (PI3K) and protein kinase B (PKB/Akt), which both regulate angiogenesis downstream of VEGFR-2, are additional targets that could be considered to achieve vascular normalisation. Selectively blocking one or more of these molecules resulted in persistent vascular changes for up to 2 weeks post treatment in human xenograft-bearing mice.^60,61^

### Rgs5: a master gene for abnormal vascular morphology in the tumour

Endothelial cells in abnormal tumour vessels are not homogeneous, and differ in phenotype from their non-tumour counterparts.^30,62^ Although transcriptomic profiles can vary between tumour vessels and normal vessels, few genes directly associated with the abnormal neovasculature have been identified.^59^ The regulator of G-protein signalling 5 (Rgs5) has been shown to be a master gene for abnormal vascular morphology in the tumour.^63^ Rgs5 is expressed in a variety of organs and upregulated on blood vessels in Rip1-Tag5 tumours. Pericytes around the vessels of these tumours were predominantly PDGFR-β positive. Crossing Rip1-Tag5 with *Rgs5*^*−/−*^ mice resulted in vascular remodelling and pericyte maturation, which was assessed by α-smooth muscle actin (αSMA) and neural/glial antigen 2 (NG2) expression. Additional observations such as reduced vascular leakage, increased perfusion and higher oxygen levels are consistent with vascular normalisation. Other tumour models crossed with the Rgs5^−/−^ mice also generated similar findings. A gene whose expression directly relates to abnormal vessel formation could theoretically become a target for therapeutic genome editing.^64^

### Promoting factors that support vessel normalisation

As well as blocking factors that are actively proangiogenic, promoting factors that initiate vascular maturation and sustain maintenance were found to normalise the vessels in a tumour. Unlike oncogenic H-Ras GTPases, R-Ras has been identified as an inhibitor of endothelial cell proliferation and functions instead as a regulator of vessel integrity and maturation during vascular remodelling to promote normalisation of tumour vasculature.^61^

Members of the family of angiopoietins have contrasting effects on the vessels. Ang-1 promotes vessel maturation and pericyte recruitment, whereas Ang-2 promotes endothelial cell death and vascular disruption and also works as an antagonist to Ang-1 via the Tie-2 receptor. Inhibiting Ang-2 while simultaneously activating the Tie-2 receptor leads to vascular normalisation in glioma and LLC in mouse models.^65,66^

Semaphorins comprise a large and diverse family of secreted, membrane-associated or membrane-bound signalling proteins that are essential for the development and maintenance of many tissues.^67^ The class 3 semaphorins Sema3A and Sema3F are assigned to the vascular system and counterbalance the actions of VEGF via the Sema3A–neuropilin receptor (NRP-1) complex. In tumour angiogenesis this balance falls in favour of VEGF, but in studies that followed the targeted delivery of Sema3A, tumour angiogenesis was inhibited and vessels regained a functional morphology.^68,69^

Nucleolin is predominantly localised in the nucleolus but is also found on the surface of proliferating endothelial cells. Its inhibition using a blocking antibody reduced tube formation and led to vessel normalisation in vivo, possibly via endothelial cell apoptosis as the levels of the anti-apoptotic molecule Bcl-2 were also reduced.^70^

Notch/Delta like ligand (DLL)1 signalling is an important determinant of cell fate in early development.^71^ In sprouting angiogenesis, Notch-1/DLL4 signalling shapes the vascular network by downregulating the VEGF-induced activation required for an endothelial cell to become a tip cell, which leads the way for proliferating stalk cells that eventually form the tubular network. Notch-1-deleted endothelial cells therefore preferentially become tip cells, which promotes an abnormal vasculature.^72,73^ The anti-malaria drug chloroquine was found to induce vessel normalisation by stimulating the Notch-1/DLL4 signalling pathway.^74,75^ In vivo, chloroquine reduced the tumour mass and improved the tumour milieu by increasing tissue perfusion, thereby lowering hypoxia and reducing tumour cell invasion while enhancing tissue sensitivity to chemotherapy.

The TME in solid cancers is acidic as a consequence of hypoxia and increased glucose metabolism. The metabolism of tumour endothelial cells (TECs), which are also hyperglycolytic, has gained attention over past years,^76^ as the normalisation of TEC metabolism is a major contributor to tumour vessel normalisation. Notably, pharmacological inhibition of tumour-cell-specific cyclooxygenase (COX)-2 leads to reduced expression of *VEGF* and, consequently, of *PFKFB3*, the gene that encodes 6-phosphofructo-2-kinase/fructose-2,6-biphosphatase 3, in the TECs. Treatment with a COX-2 inhibitor normalises glucose metabolism in TECs and inhibits PFKFB3-mediated endothelial cell motility, leading to a reduction in the formation of tip cells and filopodia and decreased branching in the neovessels.^77^ An earlier extensive study that used either a blocking agent against PFKFB3 or transgenic mice +/− knockout mice, with the aim of slowing glucose metabolism, resulted in a similar normalisation effect on the neovessels.^78^

### External factors that promote blood vessel normalisation

Tumour blood vessel normalisation can also occur without specifically targeting blocking or activating molecular angiogenic signalling factors. Cell death in response to radiation is largely dependent on the dosing and time frame of the therapy. A single high dose of radiation (30–60 Gy) is known to destroy most cells in the targeted tissue, causing a lasting decrease in vascularity.^79^ However, fractionated radiation, in which the dose is split and given over several intervals allows time for the normal cells to recover and undergo repair as shown in an orthotopic prostate xenograft mouse model of PC3-luc cells; here, the mice were treated with fractionated radiation of 2 Gy on each of 5 consecutive days over a total time of 2 weeks. Examination of the tissues at given time points (days 0, 1, 3, 7 and 14) revealed a remodelled vascular network with increased pericyte coverage and positive normalisation effects, such as increased perfusion and low hypoxia.^80^

Compelling observational evidence indicates that regular exercise synergises with anti-cancer therapy and rehabilitation. Data from preclinical studies suggest that physical activities/exercise promote tumour vessel maturity, followed by positive changes in tissue and oxygen perfusion and drug delivery and a boost to the immune response, all of which combine to improve the outcome of chemotherapy and radiotherapy in individual patients.^81,82^ When the underlying mechanisms of the influence of moderate exercise on tumour vessel normalisation were studied in detail in vivo and in vitro,^83^ the increased blood flow after exercise was seen to confer mechanical force on the endothelial cells. The resulting fluid shear stress on the vessel walls stimulates endothelial cells, triggering the production of nitric oxide and vascular remodelling mechanisms.^84^ The neovasculature of exercised tumour-bearing mice demonstrated all the signs of vessel normalisation, except for pericyte coverage. When the authors further investigated the effect of shear stress on endothelial cells, they observed that serum from exercised mice inhibited the formation of typical vascular structures by endothelial cells cultured on matrigel, suggesting that soluble angiogenesis inhibitory factors were secreted in the mouse model.^85^ Experiments on endothelial cells cultured under shear stress confirmed this observation. Subsequently, the authors discovered that the activation of nuclear factor of activated T cells (NFAT), via calcineurin, in endothelial cells is central to vascular remodelling in response to shear stress and leads to the upregulation of thrombospondin-1 (TSP-1), an inhibitor of angiogenesis. TSP-1 is essential for exercise-induced vessel normalisation.^85^

## Vascular normalisation, anti-cancer treatment and the TME

Even before systematic research into vessel normalisation began, the benefit of anti-angiogenic treatment in combination with chemotherapy and/or radiation became obvious. Patients who received bevacizumab, a humanised blocking antibody to VEGF-A, in combination with chemotherapy in clinical trials fared much better than patients who were given either treatment alone.^86^ It was originally thought that this outcome occurred because tumour cells, once starved and weakened, were more susceptible to treatment. This turned out to be only a partial explanation, because less perfused tissue tends to be hypoxic, and hypoxic tissue is ultimately more resistant to radiotherapy and chemotherapy.^27^ The main explanation for the benefit on patients was that anti-angiogenic treatment promoted vessel normalisation. Improved perfusion is overcoming physiological barriers to tissue oxygenation and makes radiation more effective owing to a decrease in hypoxia-induced radiation resistance.^28^ Reducing vessel leakiness decreases the interstitial hypertension and restoring a distinct pressure gradient leads to the deeper penetration of macromolecules (chemo-therapeutics) in the tissue and facilitates the migration of immune cells.

### A normalised vasculature boosts the tumour immune response

The immune response to cancer could be considered as a specialised case of immunity.^87^ In its early stages, a tumour engages in crosstalk with the innate immune system, and innate immune cells, such as macrophages, monocytes and dendritic cells that patrol the blood, accumulate at the neoplastic site. So why can the immune system not prevent tumour growth? In cancer, pathogen recognition triggers an acute inflammatory response, which recruits cytotoxic immune cells. These cells recognise and eliminate the more immunogenic cells, but this process selects for less immunogenic, often more aggressive, cells. As part of the TME, endothelial cells actively participate in the attraction of immunoregulatory factors, and can enhance or suppress the immune response, depending on their interactions and their expression of inflammatory cytokines.^88^ Lymphocyte trafficking, for example, is highly orchestrated: many molecular and physical factors must align to facilitate the initial and crucial step—lymphocyte rolling—in the process that enables T cells to exit the endothelium and make contact with the antigen-presenting cells (APCs).^89^ Although endothelial cells are not professional APCs, they do possess the capacity to express MHC-I and MHC-II molecules and can act like APCs.^90,91^ However, the presence of an aberrant tumour vasculature means that none of these factors can be co-ordinated.^92–94^ Consequently, the inflammatory response in a tumour no longer has the positive effect of an acute reaction to harmful stimuli but becomes a chronic inflammatory site; to quote Harold Dvorak, “tumours are wounds that never heal”.^95,96^ That may explain why a majority of patients do not benefit from anti-cancer immunotherapy in the long term. Vessel normalisation, linked to increased tissue perfusion and reduction of stromal components such as cancer-associated fibroblasts (CAFs) and collagen, to mention a few, directly promotes immune cell infiltration and functionality and as a result enhances the response to immune therapy.^97^

As indicated above, blood vessel normalisation leads to an improved immune response, involving T-lymphocyte recruitment from the secondary lymphoid organs to the tumour tissue and the accumulation of functional immune cells in the TEM,^93^ and unrestricted blood flow and restored perfusion of the tissue reduce hypoxia. Hypoxia is known to contribute to immune resistance and immune suppression in the TEM,^28,98^ as hypoxic regions are highly infiltrated by immune-suppressive cells such as myeloid-derived suppressor cells, tumour-associated macrophages and T-regulatory (Treg) cells, which are immune-suppressive.^99,100^ Vessel normalisation also restores the functionality of the luminal lining, or glycocalyx, in the neovessels, which is essential for leukocyte rolling.^101^ High levels of VEGF suppress T-cell infiltration into tumours by decreasing the expression of the T-cell-attracting chemokines CXCL10 and CXCL11; consequently, this effect is reversed upon vessel normalisation.^102^

The success of treatment using immune checkpoint inhibitors is increased in patients with a higher number of pre-existing tumour-infiltrating immune cells that express PD-L1, the ligand for programmed death-1 (PD-1).^103,104^ PD-1, alongside cytotoxic T-lymphocyte-associated protein 4 (CTLA-4) and lymphocyte activation gene 3 (LAG-3), is a well-studied inhibitory immune checkpoint that negatively regulates T-cell effector function and, by themselves synthesising these checkpoint proteins, cancer cells can avoid being attacked.^105–107^ Vascular normalisation mediated by low-dose anti-angiogenic treatment can convert cancers that are unresponsive to immunotherapy into responders, as demonstrated in a small group of patients with glioma in whom the beneficial effects of the immune checkpoint inhibitor ipilimumab were observed only in combination with bevacizumab.^108^ A combination of bevacizumab and CTLA-4 immune checkpoint inhibitors has proven favourable for the treatment of melanoma patients, possibly by evoking humoral immunity to galectin-1, an angiogenic and pro-tumour factor.^109,110^

### An improved immune response supports vessel normalisation

It also transpires that an improved immune response is not only a consequence of tumour vessel normalisation but that it also contributes to vessel normalisation. Patients with tumours that were eosinophil-rich had better overall prognoses than those with fewer eosinophils.^111^ Eosinophils contribute to tumour immunity by secreting chemoattractants for CD8^+^ T cells. The intravenous transfer of activated eosinophils into MO4 (mouse melanoma) bearing mice (in combination with activated T cells) induced a remarkable normalisation effect on the neovasculature, including enhanced pericyte recruitment and downregulation of the Rgs5 gene.^112^ Activated eosinophils altered the TME by shifting the balance of TAMs from the invasion- and metastasis-promoting phenotype, M2, to the classically activated phenotype, M1.^113,114^ A similar effect on macrophage polarisation could be achieved by inhibition of the Ang/Tie2 and the pyruvate dehydrogenase/hypoxia-inducible factor 1-α (PDH/HIF-1-α) pathways.^115,116^ CD4^+^ and CD8^+^ T cells adoptively transferred into Rgs5-negative Rip1-Tag5 mice prolonged their survival because the T cells were able to significantly infiltrate the ‘normalised’ tumours of the knockout mice but not of wild type mice.^63^ Experimentally disrupting the vessel normalisation process using an NG2-knockout mouse model (which hampers pericyte recruitment) resulted in reduced CD4^+^ T_H_1 cell infiltration. In turn, the depletion or deactivation of CD4^+^ T_H_1 cells decreases tumour vessel normalisation.^117^ T cells are known to secrete interferon γ, which is known to regulate angiogenesis.^118,119^ The normalised tumour vasculature and CD4^+^ T_H_1 cell immune response create some sort of positive feedback loop that generates a controlled angiogenic response.^120,121^

## Lessons from glioblastoma

In a clinical study in which glioblastoma patients were given a single dose of AZD2171 (cediranib, a VEGFR inhibitor) signs of vessel normalisation—based on the calculation of a ‘vascular normalisation index’—lasted for as long as 28 days, with some features of the normalised network persisting for up to 4 months. Vascular permeability/flow and microvessel volume assessed by fMRI correlated positively with the clinical outcome of patients with glioblastoma. For most patients who showed a high degree of vascular normalisation after a single dose of cediranib only, the clinical outcome improved.^122,123^ In a subset of patients with newly diagnosed glioblastoma within a Phase 2 trial that had received a regime of cediranib and chemoradiation, better perfusion and, consequently, higher oxygenation of the tumour tissue were associated with improved overall survival. Oedema in the brain was reduced and responses to radiotherapy were enhanced.^124^

The dose-dependent response to bevacizumab in the orthotopic glioma mouse model U87 showed that vessel regression and vessel normalisation occurs in animal treated with low (subclinical) and medium to high doses. Tumour regression and prolonged survival of the animals, however, was only observed in the medium to high dose group. The authors did not find bevacizumab triggered invasive behaviour of the glioma cells within their treatment time frame of 12–25 days.^125^ Although the anti-angiogenic treatment of brain tumours and accompanying vessel normalisation are associated with an overall benefit in some studies, data from other studies have revealed conflicting results. Keunen et al. reported that treatment of human glioblastoma xenografts in rats with bevacizumab decreased the number of larger sized vessels and rendered the tumour tissue more homogeneous but also increased tumour cell migration into healthy parts of the rat brain.^126^ The authors suggested that a drop in tumour oxygenation caused by fewer blood vessels triggered a metabolic change in the tumour cells that shifted them towards an invasive phenotype. Whether or not these findings translate to end-stage glioblastoma patients treated with bevacizumab is unknown.^127^

Normalisation of brain tumour vessels might be expected to help to restore the blood–brain barrier (BBB), which could be considered as an adverse effect on therapy, as this reduces the efficiency by which subsequent chemotherapy agents pass through the endothelium.^128,129^ Furthermore, gliomas are amongst a special group of cancers that are known to use vascular co-option for the growth of primary tumour sites and for metastasis. The cancer cell cytoskeleton and cell motility are fundamental factors in the molecular mechanisms of vascular co-option and mediated by Cdc42, a small Rho GTPase that regulates filopodia formation of the actin cytoskeleton and integrins.^130–132^ Integrin adhesion via the β1 chain seems essential for cancer cells to adhere to endothelial cells to both invade and escape from the vascular system through intercalation.^133,104^ In the brain, glioblastoma cells with cytoskeletal actin extensions, called flectopodia, adhere to the blood vessels by connecting in an astrocyte-like manner to the supporting pericytes suggesting that vessel normalisation and stabilisation by pericytes could potentially support vascular co-option and hence metastasis.^134^ However, a strong link between vascular co-option and vessel normalisation after anti-angiogenic treatment needs to be determined and the potential for therapeutically targeting vascular co-option evaluated.

## How can we seize the moment?

Most FDA-approved anti-angiogenic drugs are primarily given when standard first-line cancer treatment has failed or when a patient is diagnosed with an advanced stage of the disease (see Fig. 4). We also know that, in principle, it is possible to use these agents to create optimised conditions in the TME to push the therapeutic impact of other approaches further. We thus need to be able to identify this ‘window of opportunity' by scrutinising the temporal changes in the TME as cancers evolve so that we define or at least predict the providential moment to for treatment.

### Predicting a response to vessel normalisation treatment

Clinical trials involving breast cancer patients who received a single dose of bevacizumab followed by chemotherapy showed that the microvessel density prior to bevacizumab treatment could be used as a marker to predict treatment outcome—only those women whose tumours were highly vascularised before the treatment responded to bevacizumab-induced vessel normalisation.^135^

The genetic composition of tumour cells can determine whether or not a patient will respond to treatment by undergoing tumour vessel normalisation. So-called ‘Good Prognosis Angiogenesis Genes’ (GPAGs), discovered by screening breast cancer gene databases, are predominantly associated with cell–cell adhesion and smooth-muscle cell proliferation, and such gene expression programs are common in pericytes and pericyte recruitment in vessel normalisation GPAGs are also associated with immune response pathways, especially T-cell receptor signalling.^117^ This association underscores the potential to use gene expression signatures associated with pericyte function to predict not merely vessel normalisation but perhaps also the efficacy of an associated immune response. As such it exemplifies the intimate relationship between vascular function and the immunological state of cancers.

### Determining the window of normalisation

In humans, non-invasive monitoring down to the resolution of microvessels still remains a challenge. MRI, computed tomography (CT) and positron emission tomography (PET) scans are imaging methods that are routinely used in the clinic to measure the size and location of tumours and, although large vessel such as arteries and veins can be seen using a contrast agent (magnetic resonance angiogram or ‘MRA’), the spatial resolution is too low for microvessels, and the movement of fluids, essentially blood, cannot be detected. The clinical use of fMRI, which allows the measurement of blood oxygenation relative to the paramagnetic properties of deoxyhaemoglobin also lags behind its use in research.^136^ In animal models, however, good results were obtained monitoring vessel normalisation using, for example, imaging biomarkers with fMRI or by photoacoustic imaging. Both methods were able to denote the normalisation window in mice and rats after low-dose anti-angiogenic treatment.^47,48,137^ The practical advantages of photoacoustic imaging over approaches that demand large and expensive instruments renders this technique a likely choice for monitoring the (tumour) vasculature in humans and in the clinic in the near future.^138^

A further approach to determine the beginning and end of the vessel normalisation process involves non-invasive imaging of the tumour redox state and energy metabolism. Monitoring the partial pressure of oxygen (pO_2_) in murine tumours using electron paramagnetic resonance (EPR) and fMRI imaging identified a normalisation window 2–4 days after anti-angiogenic treatment with sunitinib. The perfusion of the tissue was improved and reduced the occurrence of acute hypoxia. The oxygen levels in the tissues were stabilised to normal and an oxidative shift of the tumour redox status in the metabolic pyruvate/lactate flux measured by an exogenous nitroxide probe and ^13^C-NMR was revealed.^31,139^ In principle, it would be possible to measure metabolic changes using PET—for example, fluorodeoxyglucose (FDG)-PET could be used to measure glucose uptake in endothelial cells. A reduced glucose uptake can lead to vessel normalisation and hence be a valuable indicator of this process.^77,78,140^ At the same time, a widespread goal is to step away from costly and time-consuming methods towards strategies that are more amenable to routine clinical practice.

Few molecular markers that indicate the presence of a normalised TME have been identified. A potential candidate, however, is apelin, a peptide ligand for the apelin receptor (APJ), which, when activated, regulates blood pressure and encourages the formation of new blood vessels.^141^ The expression of apelin is driven by hypoxia and hence is comparably high in cancers.^142^ Low apelin mRNA or protein serum levels were shown to indicate vessel normalisation in the mouse model; here, the lowest apelin levels were seen around day 5 after treatment, suggesting a normalisation window between days 3 and 5.^143^

## Outlook

So much evidence now exists for the benefit of a normalised tumour vascular network as part of any cancer treatment that it offers an extraordinary opportunity to improve treatment outcomes. Immunotherapy, for instance, has progressed rapidly in the past five years, but not all types of tumour respond to this approach; it is now therefore vital to complement this treatment, and others, with well controlled adjustments to the vasculature. Finding the opportune moment to normalise the vasculature remains a challenge, and the discovery of a reliable and universally measurable indicator has yet to be made. However, some approaches to assess the problem are emerging. Measuring hypoxia-driven biomarkers, such as apelin, in a blood test might be more feasible than measuring the expression levels of a protein like CD109 in dormant endothelial cells or assessing blood oxygenation by fMRI each day over weeks.^144^

An additional challenge lies in how best to create such a ‘window of opportunity’ therapeutically and to keep it open for as long as necessary. Systemic and oral anti-angiogenic treatments can come with severe side effects. The range of clinically approved anti-angiogenic medication is limited, and most anti-angiogenic drugs will be given after conventional treatments have failed or if a patient is diagnosed with advanced stages of cancer (see Fig. 3). Both factors can be restrictive in the design of combination therapy. Applying precision radiotherapy in early stages to create vessel normalisation has considerable potential. Endothelial cells are, by definition, normal cells, albeit heterogeneic, and are more efficient in their DNA repair than tumour cells. Consequently, precision radiotherapy can promote a reduction in angiogenic factors through the death of cancer cells whilst permitting the surviving endothelial cells to relax and remodel under normalised angiogenic conditions. This vascular reset in turn can serve as a base for almost any other subsequent treatment intervention.

Whereas the normalisation effect achieved by medication or radiotherapy can be short-lived and expensive, physical activity is cost-effective and discretionary, virtually free of side effects and applicable over extended periods of time. The benefit of creating a vessel normalisation window simply through moderate aerobic activity would be undeniable. The rate of activity that yielded promising results in exercising mice in a laboratory setting would translate to a daily brisk walk in humans, which is achievable even under life-changing circumstances.^81^ Physical activity during or after cancer treatment has long been associated with overall well-being and improved quality of live. Now biological evidence can be added that increased blood flow will aid any following treatment and should be explored further. Nearly 2000 clinical trials are currently actively studying the behavioural impact of exercise on cancer patients, and valuable data can be obtained from these studies (Refer to https://clinicaltrials.gov/).

In conclusion, cancer researchers are increasingly appreciating that only a complete understanding of tumour plasticity and the evolutionary biology supporting tumorigenesis will achieve durable treatment responses,^145^ with evidence indicating that in-depth analyses of the vascular architecture and the dynamics in vascular function will provide the most robust indications regarding tumour adaptation. The classification of evolutionary stages in cancer would allow, for instance, a range of approved end-stage cancer therapies to be administered to patients at earlier stages of cancer without raising the risk for side effects.

## Data Availability

As a review article access to all associated primary data relating to the article is the responsibility of the authors of the cited primary research. The review article does not contain any primary data.
